# Stigmasterol Simultaneously Induces Apoptosis and Protective Autophagy by Inhibiting Akt/mTOR Pathway in Gastric Cancer Cells

**DOI:** 10.3389/fonc.2021.629008

**Published:** 2021-02-23

**Authors:** Huange Zhao, Xian Zhang, Min Wang, Yingying Lin, Songlin Zhou

**Affiliations:** ^1^ Key Laboratory of Tropical Translational Medicine of the Ministry of Education & Hainan Provincial Key Laboratory of Tropical Medicine, Hainan Medical University, Haikou, China; ^2^ Schools of Basic Medicine and Life Sciences, Hainan Medical University, Haikou, China

**Keywords:** stigmasterol, apoptosis, autophagy, Akt/mTOR signaling pathway, gastric cancer cells

## Abstract

**Background:**

Stigmasterol (SS) has been proven to possess potential anticancer activities in several cancer cell lines, but its molecular mechanism is still unknown. Thus, we investigated whether SS has the capabilities of inducing autophagy and its molecular mechanisms in gastric cancer cells.

**Methods:**

We used CCK8 assay, clone formation assay, and EdU proliferation assay to assess the effects of SS on cell proliferation in SGC-7901 and MGC-803 cells *in vitro*, and its inhibition on the tumor growth of gastric cancer was observed *in vivo*. Apoptosis induced by SS was demonstrated using Hoechst and TUNEL staining, annexin V-FITC/PI assay. Immunofluorescence staining is used to detect the formation of autophagosomes triggered by SS. Apoptosis and autophagy related proteins were analyzed by western blot.

**Results:**

The results indicated that SS treatment inhibited cell proliferation in SGC-7901 and MGC-803 cells. Furthermore, SS treatment induced apoptosis and autophagy by blocking Akt/mTOR signaling pathway. The pretreatment with the Akt inhibitor MK-2206 could promote apoptosis and autophagy induced by SS, predicting that Akt/mTOR pathway is involved in SS-induced apoptosis and autophagy. In addition, blockade of autophagy with 3-MA (an inhibitor of autophagy) enhanced SS-induced apoptosis in SGC-7901 and MGC-803 cells, implying that autophagy mediated by SS plays a cytoprotective role against apoptosis. Finally, an *in vivo* study demonstrated that tumor growth of gastric cancer was suppressed by SS in a xenograft model.

**Conclusion:**

Our findings illustrate for the first time that SS simultaneously induces apoptosis and protective autophagy by inhibiting Akt/mTOR pathway in gastric cancer cells, and SS may become a potential anticancer drug in treating gastric cancer in the future.

## Introduction

Plant sterols are the particular phytochemicals that have various pharmacological activities, such as antidiabetic, anti-inflammatory, and anticancer (1, 2). Additionally, it has been suggested that the intake of phytosterol-rich foods may lower the risk of cancer by 20 percent (3–5). Stigmasterol [(C_29_H_48_O), SS] is the common plant sterol in various foods and vegetables. In recent years, SS has received more and more attention for their anticancer properties against various cancer cell lines by different mechanisms, including inhibition of tumor cell growth (6), suppression of tumor angiogenesis and cholangiocarcinoma growth (7), and induction of cancer cell apoptosis (8–12). Several studies have been conducted in the past on apoptosis induced by stigmasterol. However, in view of the crosstalk between autophagy and apoptosis, studies devoted to mechanism of action of stigmasterol seem to be far from adequate.

Apoptosis and autophagy are two highly regulated processes that are essential for human homeostasis (13). Apoptosis is a process of programmed cell death. Apoptotic cells show a series of morphologic changes, including cell shrinkage and blebbing, chromatin condensation, and nuclear fragmentation (14). Autophagy is normally a cell-survival process in which the obsolete, damaged, or harmful macromolecular assemblies are degraded and then recycled. However, excess or intense autophagy may cause type II cell death (15). Most chemical agents exert their antitumor activities through inducing cell apoptosis both *in vitro* and *in vivo* (16–18). Therefore, effective induction of tumor cell apoptosis is the most important character for a new chemical agent for translation use in cancer treatment. There is growing evidence suggesting that autophagy can cause cell death by collaborating with apoptosis in some cellular stress conditions such as nutrition-deprived or hypoxic environments (19, 20). However, the relationship between autophagy and tumorgenesis is inconsistent, and both suppressive and protective activities have been reported (21, 22). In this regard, it is urgently needed to investigate extensively the coordinative or crosstalk mechanisms of apoptosis and autophagy. A comprehensive understanding of the relationship between apoptosis and autophagy is important for the development of effective cancer therapeutics.

The Akt/mTOR signaling is a classic regulating pathway, which is normally an inactive state and inhibits autophagy process (23, 24). Thus, inhibition of Akt/mTOR signaling by small molecules or chemical agents triggers the autophagy process. In addition, several studies have demonstrated that Akt/mTOR signaling pathway is involved in promotion of apoptosis and autophagy process (25–27). Nevertheless, few studies have elucidated the molecular mechanism of SS in apoptosis and autophagy on human cancer cells. Therefore, in this study, we confirmed that SS could simultaneously induce apoptosis and protective autophagy in two gastric cancer cell lines by blocking the Akt/mTOR signaling pathway. SS-induced autophagy plays a protective role in gastric cancer cells. Our results suggest that SS combined with autophagy inhibitors may be a more reasonable treatment strategy for gastric cancer.

## Materials and Methods

### Reagents and Antibodies

Stigmasterol (purity≥98%) was purchased from Sigma-Aldrich (St. Louis, Missouri, USA). Cell counting Kit-8, Hoechst 33258, one step TUNEL Apoptosis Assay Kit, Annexin V-FITC Apoptosis Detection Kit were obtained from Beyotime Institute of Biotechnology (Nantong, China). Caspase-3, cleaved caspase-3, PARP, cleaved PARP, Bax, Bcl-2, and the secondary antibodies were purchased from Abcam (Cambridge, MA, UK). Anti-LC3 was purchased from MBL Beijing Biotech Co. Ltd (Beijing, China). Akt, p-Akt, mTOR, p-mTOR, beclin-1, p62 were purchased from Cell Signaling Technology (Bosson, MA, USA).

### Cell Culture and Treatment

Human gastric cancer cell line SGC-7901, MGC-803, and normal GES-1 cell line were purchased from Type Culture Library, Chinese Academy of Sciences, Shanghai Instituted of Cell Biology (Shanghai, China). Cells were cultured in RPMI 1640 medium containing 10% fetal bovine serum at 37°C and in a 5% CO_2_ humidified atmosphere. Cells were then incubated with different dose of SS (0, 10, 20 µM) for 48 h before being collected in the logarithmic phase. We repeated all experiments at least three times.

### Cell Viability Assay

Viability of cells was measured with CCK-8 kit follow the manufacturer’s instructions. Cells were harvested by trypsinization at the logarithmic growth phase and seeded in 96-well plate at density of 0.2×10^4^ cells/well in triplicate for 48 h, and inoculate with different concentrations of SS (2.5, 5, 10, 15, 20, 25, 30 µM) or without addition of SS for 24, 48, and 72 h. Then, 10 µl CCK-8 was added to each well and the cells were further incubated for 1.5 h at 37°C in 5% CO_2_. Bio-Tek EXL808 microplate reader was used to read the absorbance of each well at 450 nm (Winooski, VT, USA).

To observe the capacity changes of single cells to form a colony, cells were inoculated with corresponding dose (0, 10, 20 µM) of SS for 48 h. The fresh RPMI1640 medium was used every 3 days to replace the medicated culture medium, and cells were cultured for 10 days. Then, the cellular colonies were immobilized, treated with crystal violet solution and the numbers of colonies were counted.

Cells were seeded in a confocal fluorescence microscope special dish and treated with indicated dose of SS (0, 10, 20 µM) for 48 h. Cells proliferation was tested using EdU Cell Proliferation Kit with Alexa Fluor 594 in accordance with the operating instructions’ for processing. Cell nuclei were co-stained with Hochest 33258. After that, EdU-positive cells were monitored with a fluorescence microscope (Olympus Corporation, Tokyo, Japan).

### Apoptotic Cell Morphology Assay

Apoptotic cell morphology was measured by Hoechst 33258 and TUNEL staining. Briefly, gastric cells were cultured in a co focal fluorescence microscope special dish and treated with indicated concentrations of SS (0, 10, 20 µM) for 48 h. Then, morphological changes of gastric cancer cells were monitored *via* a fluorescence microscope (Olympus Corporation, Tokyo, Japan). Morphological apoptotic cells were calculated in 10 random fields.

### Apoptosis Assay

Early and late apoptotic cell changes induced by SS were determined by Annexin V-FITC/PI apoptosis detection kit. Briefly, cells were cultured with the indicated dose of SS for 48 h, then, cells were collected in the same tubes. After centrifugation, the cells suspended in Annexin V-FITC binding buffer and were dyed with FITC Annexin V and PI in the dark. Percentages of apoptotic cells were immediately assessed by flow cytometry using the cell quest software (BD Accuri C6, USA). Total apoptotic rate was calculated by combining early and late apoptotic cells.

### Autophagy Was Measured by Immunofluorescence Staining

Cells cultured on a disinfectant coverslips at a density of 0.5×10^5^ cell/ml overnight. After cells were treated with SS for 48 h, cells were rinsed, immobilized, infiltrated, and covered with 5% skim milk. After rinsing with PBS, cells were incubated with LC3-II antibody at 4°C overnight. Following this, cells were hatched with second antibody and were co-dyeing with Hoechst 33258 solutions. LC3 green fluorescent puncta formations were observed with a confocal laser-scanning microscope (Olympus Corporation, Tokyo, Japan). The LC3 puncta were quantified using ImageJ quantification tool. The average number of LC3 puncta was counted from 10 different fields under each condition.

### Western Blot Assay

The proteins were lysed in a lysate buffer containing the protease inhibitor RIPA and the concentration of protein was quantified using BCA kit. The same amount of protein was isolated by SDS-PAGE gels and transferred to the PVDF membrane, hatched with 5% skim milk in PBST to block non-specific antigen and were labeled with various primary antibodies against corresponding antigen at 37°C for 2 h. After rinsing three times, the membrane was bathed with secondary antibody for 1 h at 37°C, proteins blot were visualized with an enhance chemiluminescence detection kit (Thermo Scientific, Rockford, IL, USA). The β-actin was selected as the internal reference.

### Tumor Xenograft to Assess Antitumor Activity

Nude Balb/c mice were purchased from Changsha Tianqin Biotechnology Co. LTD. All animal experiments are approved by the Animal Care and Use Committee of Hainan Medical College and in accordance with animal rules (grant number: HYLL-2020-026). In brief, the nude mice were subcutaneously inoculated with the corresponding cell lines (SGC-7901 or MGC-803) with of 2×10^7^ cells/ml in the right inguinal region. When tumor volumes reached approximately 100 mm^3^ (about 7 days injection), the mice were allocated randomly into two groups (five mice/group). Control group mice were injected with saline and mice from the experimental group injected with SS through the tail vein every 3 days, mice received seven-injection total. Tumor volumes and weight were detected every 4 days; mice were euthanized on day 28. The tumor volume (V) was calculated using the equation: V = (length × width^2^)/2.

### Statistical Analysis

All experiments data were exhibited as the mean ± SD in triplicates. Student’s t-test was performed to compare statistical differences between two groups and one/two-way ANOVA was used to compare data among different groups employing the GraphPad Prism software. P*< 0.05, p**< 0.01, p^#^< 0.05, p^##^< 0.01 was thought to be statistically different.

## Results

### SS Inhibits Viability and Proliferation of Gastric Cancer Cells

**Figure 1:**
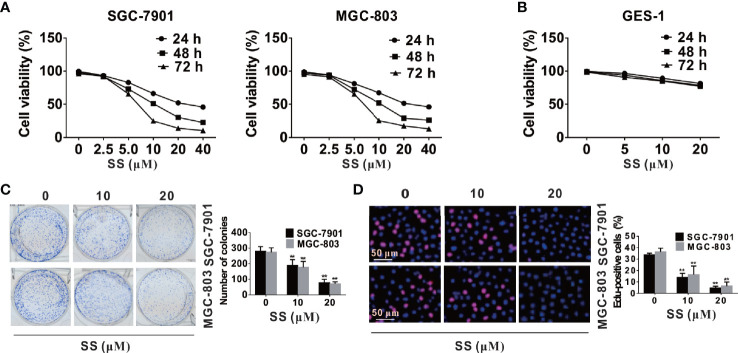
Stigmasterol (SS) inhibits viability and proliferation of gastric cancer cells. **(A, B)** Gastric cancer cells and normal cells were treated with indicated concentrations (2.5, 5, 10, 15, 20, 25, 30 µM) or without addition of SS for 24, 48, and 72 h, and the cell viability was evaluated by CCK8 assay. **(C)** SGC-7901 and MGC-803 cells were incubated with 0, 10, 20 µM SS for 10 days, and the number of colonies were calculated. **(D)** Cells proliferation was tested using EdU Cell Proliferation Kit with Alexa Fluor 594, and EdU-positive cells were monitored in five random fields/sample. Data are expressed as mean ± SD of triplicate experiment. **P < 0.01 *vs.* control (0 µM SS).

To investigate whether SS could inhibit cell growth in SGC-7901 and MGC-803 cells, a series of concentrations of SS (0, 2.5, 5, 10, 15, 20, 25, 30 µM) were used to treat SGC-7901 and MGC-803 cells, and detect the cell viability at different time points (24, 48, and 72 h) by CCK-8 assay. The results show that SS significantly inhibited cells viability in a time- and dose-dependent manner in SGC-7901 and MGC-803 cells (**Figure 1A**). But SS treatments did not influence the viabilities in normal GES-1 cells (**Figure 1B**), suggesting that the effect of SS is specific to the gastric cancer cells. Consistently, the inhibition of cell growth by SS was tested by cloning formation and EdU incorporation assays. The number of colonies in the SS treated cells was significantly decreased with the increase of SS concentrations (**Figure 1C**). As predicted, significantly lower percentage of EdU-positive cells were found in SS-treated cells (**Figure 1D**). Taken together, these data suggest that SS could inhibit cell growth and proliferation in SGC-7901 and MGC-803 cells. In addition, the results in **Figure 1B** also show that minimal effective concentration of SS is 5 µM, and the peak concentration is 40 μM. To simplify the study, the SS concentrations, *i.e.*, 10 and 20 μM, were selected for the subsequent experiments.

### SS Induces Apoptosis in Gastric Cancer Cells

**Figure 2:**
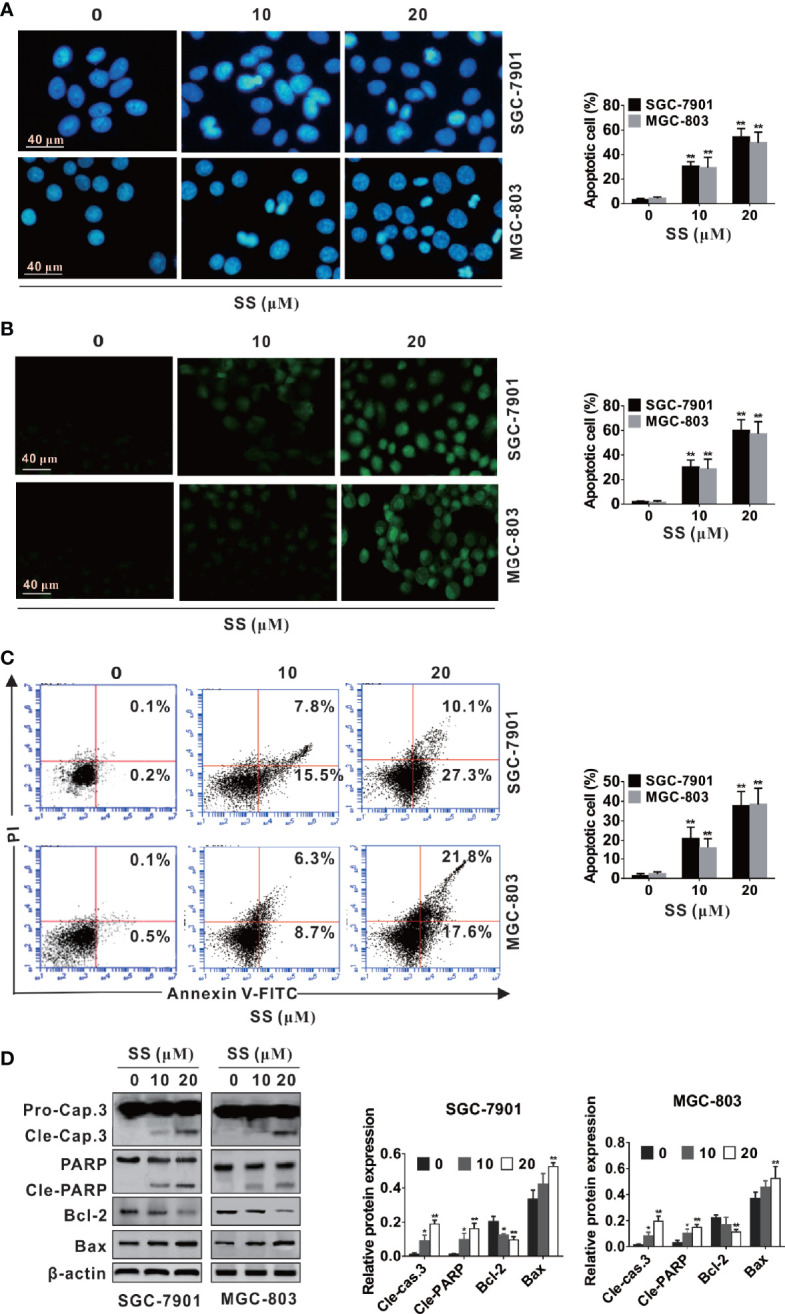
Stigmasterol (SS) induces apoptosis in gastric cancer cells. SGC-7901 and MGC-803 cells were treated with indicated dose or without addition of SS for 48 h. **(A, B)** Cells were stained with Hoechst 33258 and TUNEL, and morphological changes of gastric cancer cells were monitored under a fluorescence microscope. **(C)** Early and late apoptotic cells changes induced by SS was determined by Annexin V-FITC/PI. **(D)** The expression of caspase-3, cleaved caspase-3, PARP, cleaved-PARP, Bcl-2, Bax, and β-actin were measured by western blot analysis. Data are expressed as mean ± SD of triplicate experiment. *P < 0.05, **P < 0.01 *vs.* control (0 µM SS).

To determine whether the inhibitory proliferative effect of SS in SGC-7901 and MGC-803 cells was associated with apoptosis, cell morphology change in cells was observed using Hoechst and TUNEL staining. As shown in **Figure 2A**, typical apoptotic morphological characteristics such as nuclear fragment and chromatin condensation were significantly increased after cells were incubated with SS. Meanwhile, the percentage of apoptotic cells was increased with the increase of SS concentration. In addition, the TUNEL fluorescence spot in the SGC-7901 and MGC-803 cells was brighter than that in the control group and the number of TUNEL-positive cells was significantly increased (**Figure 2B**).

To further confirm the ability of SS-induced apoptosis in cells, the number of apoptotic cells (both early and late stage) was calculated by apoptosis assays kit. As shown in **Figure 2C**, both early and late apoptotic cells were increased with the increase of SS concentration. The increase of apoptosis rate was parallel to the increase of concentration. These results proved that SS had the ability to trigger cell death in human gastric cancer cells.

**Figure 3:**
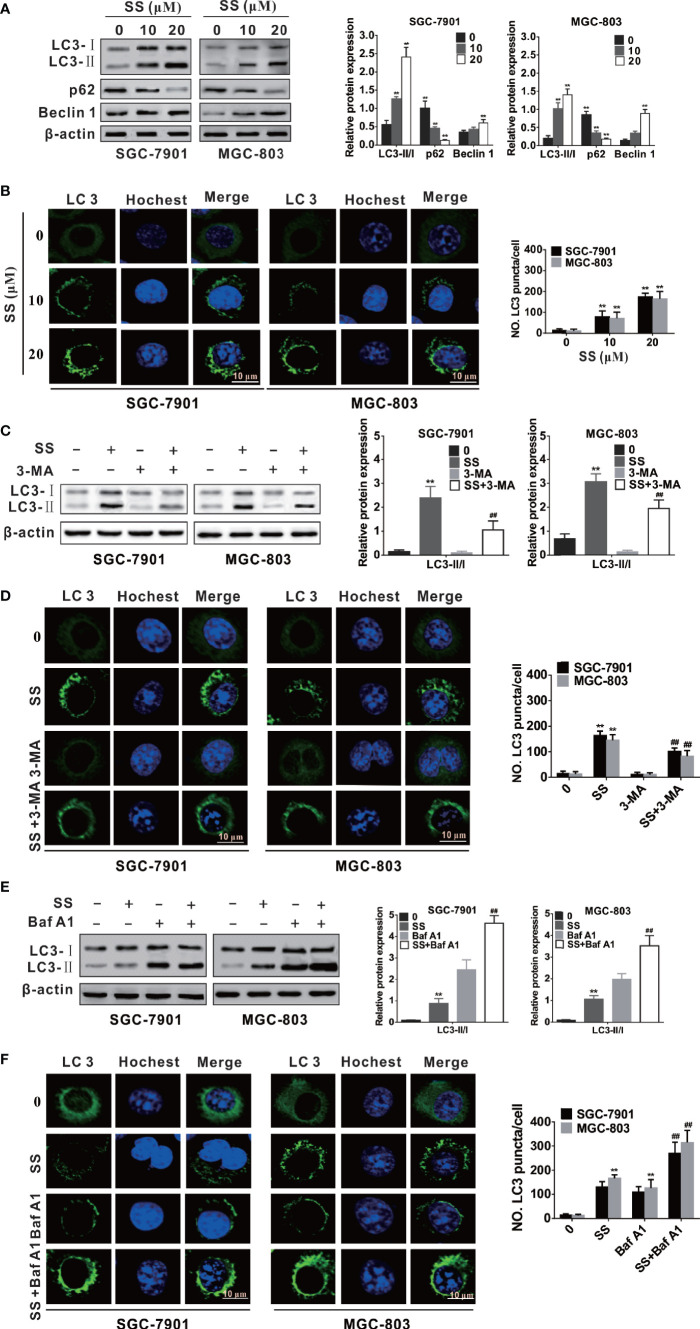
Stigmasterol (SS) induces autophagy in gastric cancer cells. **(A)** Cells were exposed to selected dose of SS and autophagy related proteins such as LC3, Beclin-1, and p62 were detected by western blot analysis. **(B)** Cells were treated as in A and stained with ant-LC3 antibody for observe of LC3 puncta under a confocal microscopy. **(C)** SGC-7901 and MGC-803 cells were treated with or without SS (20µM) in combination with or without Baf A1 (100 nM) for 48h, the expression of LC3-I, LC3-II, and p62 were detected by western blot. **(D)** Cells were treated as in C and the LC3 green fluorescent puncta formation in cytoplasm was monitored. **(E)** SGC-7901 and MGC-803 cells were treated with SS (20µM) in the absence or presence of Baf A1 (100 nM) for 48h, and the expression of LC3-I, LC3-II were detected by western blot. **(F)** Cells were treated as in **(E)** and the LC3 green fluorescent puncta formation in cytoplasm was monitored. Data are expressed as mean ± SD of triplicate experiment. *P < 0.05, **P < 0.01 *vs.* control (0 µM SS). **^#^**P < 0.05, **^##^**P < 0.01 *vs.* SS treated group.

Caspase proteins and Bcl-2 family play crucial role in the process of apoptosis (28, 29). Thus, we observed the effects of SS on apoptosis associated proteins. As shown in **Figure 3B**, SS-induced apoptosis was confirmed by the upregulated expression of Bax, cleaved caspase-3 and cleaved PARP, and the downregulated expression of Bcl-2 in a concentration-dependent manner in SGC-7901 and MGC-803 cells. These data suggested that SS induced apoptosis in gastric cancer cells.

### SS Induces Autophagy in SGC-7901 and MGC-803 Cells

Mounting evidence has indicated that many anticancer compounds have the ability to induce autophagy in different cancer cells (24, 30). In order to explore whether SS triggered autophagy in gastric cancer cells, we first detected the expression of autophagy-associated proteins, such as LC3, Beclin-1 and p62. We traced the conversion of LC3-I to LC3-II, a marker for autophagic vesicles and autophagic activity. The conjugation of autophagy-associated proteins with phosphatidylethanolamine and subsequent attachment to LC3-I forms LC3-II - making LC3-II one of the most important markers for evaluating the level of autophagy (31). As shown in **Figure 3A**, there was a concentration-dependent increase in the LC3-II form in the SS treated cells. Beclin 1 is an upstream molecule required for autophagosome formation, and plays a significant role in the induction of autophagy (32). Here, the expression of Beclin-1 was increased with the increase of concentration of SS (**Figure 3A**). It is well known that p62 protein as an ubiquitin-binding receptor protein binds directly to LC3 and is degraded by autophagy (33). Therefore, we also checked the expression of p62, as shown in **Figure 3A**, there was a dose-dependent decrease of p62 in treated cells compared with the control. Since the expression of LC3-II is related to the number of autophagosomes and autolysosomes, the quantification of LC3 positive spots is considered to be the gold standard method for evaluating the number of autophagosomes in cells (34). Here, the autophagosome formations in cytoplasm were observed in SS-treated cells under a confocal laser scanning, suggesting that SS could induce the LC3 green fluorescent puncta formation in cytoplasm and the number of LC3 green fluorescent puncta was increased when SS concentration was increased (**Figure 3B**).

In this study, considering that 3-methyladenine (3-MA) and bafilomycinA 1 (Baf A1) could block early and late phases of autophagy, respectively, gastric cancer cells were incubated with SS, and 3-MA or Baf A1, to further confirm SS-induced autophagy. Since 3-MA inhibits autophagy by blocking autophagosome formation *via* the inhibition of class III PI3K (35), it was found that 3-MA could decrease the production of LC3-II and the LC3-II green fluorescent puncta number in cells in **Figures 3C, D**. It is reported that Baf A1 inhibits lysosomal degradation of autophagosome and leads to increased accumulation of LC3 II (36). As shown in **Figures 3E, F**, when cells were co-stimulated by SS and Baf A1, the SS-induced LC3-II accumulation and the LC3-II green fluorescent puncta number in cytoplasm were more than that of cells stimulated by SS alone. Thus, autophagy indeed occurred in SS treated gastric cancer cells.

### SS Induces Autophagy and Apoptosis by Inhibiting the Akt/mTOR Pathway

**Figure 4:**
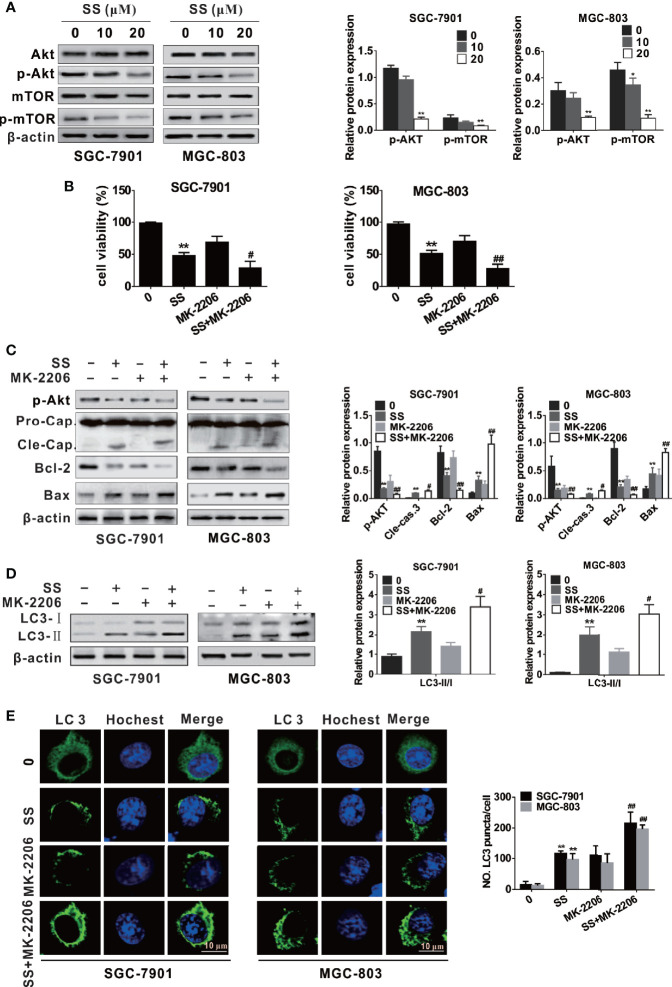
Stigmasterol (SS) induces apoptosis and autophagy by inhibiting the Akt/mTOR pathway in gastric cancer cells. **(A)** The expression levels of AKT, p-AKT, mTOR, p-mTOR, and β-actin were measured by western blot. **(B)** Cells were treated with or without SS (20 µM) in combination with or without MK-2206 (8 mM), the cell viability was detected using CCK-8 assays. **(C)** Cells were treated as in B, the phosphorylated level of AKT and apoptotic related protein cleaved caspase-3, Bcl-2, Bax, and β-actin were determined by western blot. **(D)** Cells were treated as in B, the autophagy related protein LC3 and β-actin were determined by western blot. **(E)** Cells were treated as in B, and the LC3 green fluorescent puncta formation in cytoplasm was monitored. Data are expressed as mean ± SD of triplicate experiment. **P < 0.01 *vs.* control (0 µM SS). **^#^**P < 0.05, **^##^**P < 0.01 *vs.* SS treated group.

More and more reports have suggested that Akt/mTOR signaling pathway is involved in drug-induced apoptosis and autophagy [21, 22, and 24]. To elucidate the underlying mechanism of SS-mediated autophagy and apoptosis in gastric cancer cells; we examined the level of phosphorylation of Akt and mTOR by western blot. As shown in **Figure 4A**, cells treated with SS inhibited the phosphorylation level of Akt and mTOR in a concentration-dependent manner, while the expression of total Akt and mTOR was not significantly changed.

For further confirm whether the Akt/mTOR pathway participates in SS-triggered apoptosis and autophagy in gastric cancer cells, we treated cells with SS and MK-2206 (an Akt inhibitor). As shown in **Figure 4B**, cells stimulated with SS and MK-2206 enhanced inhibition of cell viability compared with those treated with SS or MK-2206 alone. The expression of cleaved caspase-3, Bax, and LC3-II was increased and the levels of p-Akt and Bcl-2 were decreased when cells were treated with SS and MK-2206 (**Figures 4C, D**). In addition, the results of immunofluorescence are consistent with the above results (**Figure 4E**). The results demonstrated that SS induces autophagy and apoptosis by inhibiting the Akt/mTOR pathway.

### SS Induces a Protective Autophagy in Gastric Cancer Cells

The role of autophagy in cancer cells has a cytotoxic or cytoprotective function depending on the distinct cellular stress and drugs treatment (21, 22). Thus, autophagy may play the role of tumor suppressor or tumor promoter. To reveal the role of autophagy in the apoptosis process triggered by SS in gastric cancer cells, we used 3-MA to block autophagy.

**Figure 5:**
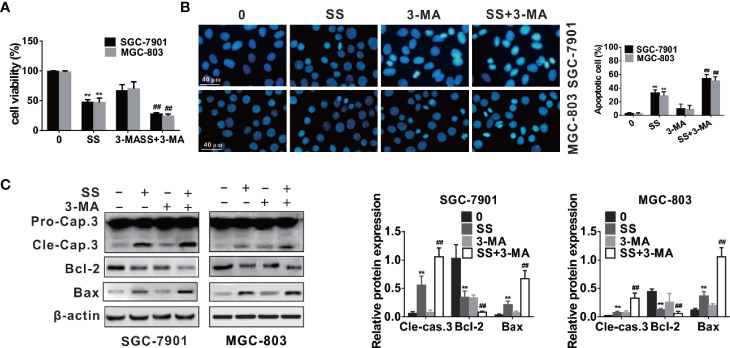
Inhibition of autophagy enhanced stigmasterol (SS) induced apoptosis in gastric cancer cells. Cells pretreated with 3-MA (5 mM), and then incubated with or without SS (20 µM) for 48 h. **(A)** Cell viability was observed by CCK-8 assay. **(B)** Morphological changes of gastric cells were monitor under a fluorescence microscope. **(C)** The expression of cleaved caspase-3, Bcl-2, Bax, and β-actin were determined by western blot. Data are expressed as mean ± SD of triplicate experiment. **P < 0.01 *vs.* control (0 µM SS). **^##^**P < 0.01 *vs.* SS treated group.

As shown in **Figure 5A**, CCK-8 assay indicated that cell viabilities were weakened in the gastric cancer cells after autophagy inhibition, and the cell typical apoptotic morphology became more pronounced when cells were treated with 3-MA and SS (**Figure 5B**). Western blot analysis also demonstrated that autophagy inhibitors increased the expression of apoptotic proteins (cleaved caspase-3 and Bax) and decreased the expression of Bcl-2 compared to those treated with 3-MA or SS alone (**Figure 5C**). All results suggested that inhibition of autophagy could enhance SS-induced apoptosis in gastric cancer cells. In other words, autophagy played a protective role against SS-induced apoptosis in gastric cancer cells.

### SS Inhibits Tumor Growth of Gastric Cancer Cells *In Vivo*

**Figure 6:**
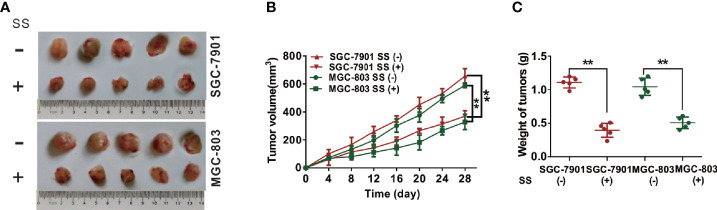
Stigmasterol (SS) inhibits gastric tumor growth *in vivo*. **(A)** Images of gastric tumors dissected from mice after treatment with indicated therapy. **(B)** The tumor volumes were detected every 3 days for 28 days. **(C)** The tumor weight was determined after mice treatment with indicated therapy. **P < 0.01 *vs.* control (0 µM SS).

To further examine the role of SS in SGC-7901 and MGC-803 cells *in vivo*, we inoculated SS into BALB/c-nude mice to set up a xenograft model and determined the tumor-related parameters. Tumor volume decreased after 8 days of treatment compared to that in control group mice (**Figures 6A, B**). Moreover, the tumor weight in SS-treated mice was also significantly reduced compared to control group mice (**Figure 6C**). Taken together, these results suggested that SS could effectively delay SGC-7901 and MGC-803 cells growth *in vivo*.

## Discussion

Gastric cancer is one of the most common cancers with its substantial morbidity and mortality around the world (32). The chemotherapeutic drugs for gastric cancer are currently used in clinical practice with adverse effects. Therefore, it is necessary to explore effective anticancer drugs with fewer side effects. SS is the main plant sterol in dietary plants with minimal side effect and low cost. Recent reports show that SS has anticancer abilities against some cancer cell lines (6–12), which provides a new strategy for cancer treatment. However, there are few studies about the effect of SS on the induction of apoptosis and autophagy, and the underlying molecular mechanisms in gastric cancer cells. Here, our findings indicated that SS could induce apoptosis and autophagy by blocking Akt/mTOR pathway, and autophagy played a protective role against SS-induced apoptosis in gastric cancer cells.

Accumulating evidence has documented that many chemotherapeutic and anticancer drugs can induce autophagy in a series of cancer cell lines, and targeted autophagy may be a promising strategy for cancer therapy (24, 30). However, there is insufficient information on the autophagy of SS in cancer cells. In this study, SS-induced autophagy in gastric cancer cells was found for the first time. The induction of SS could trigger autophagosome formation, promote the conversion of LC3-I to LC3-II, and regulate the expression of autophagy-related proteins. Autophagy and apoptosis are two catabolic pathways that maintain homeostasis, both of which are associated with the death of cancer cells. The autophagy exerts contradictory role in tumor development when cells are exposed to different stimuli and stages (19, 20). Therefore, the understanding of the relationship between apoptosis and autophagy can provide a novel scheme for the treatment of cancers. Our study manifested that SS induced apoptosis and autophagy in gastric cancer cells. Blocking autophagy with 3-MA enhanced the apoptosis induced by SS, indicating that autophagy played a cytoprotective role in SS-triggered apoptosis in gastric cancer cells. Interestingly, our result is inconsistent with the report by Sun et al. (37), which demonstrated that SS treatment inactivates autophagy in the brain tissue following ischemic/reperfusion injury. The reason of the differences could be summed up as follows. First of all, the experimental background is different. Our result was performed *in vitro* while the experimental result from the study by Sun *et al*. was obtained using animal models. Second, the cell lines that SS acts on are different. The gastric cancer cell line was selected in our experiment while Sun *et al*. studied the effect of SS on neuronal cells, which may cause a big difference of SS in function because of differences in genetic background of experimental models. Third, different drug dosages and treatment times may also produce different experimental results. Therefore, it is of value to further study the functions of SS in a series of cell lines.

The Akt/mTOR pathway is a key signaling cascade that is activated in a variety of cancers, and its activation can prompt cancer development (38, 39). In the Akt/mTOR pathway, mTOR is a significant negative regulator of autophagy and suppresses the formation of autophagosome through the activation of PS6K. Also, the phosphorylation of Akt is a significant event in the apoptosis process (40). Their continuous stimulation plays an important role in the maintenance of malignant tumors. Several anticancer drugs can induce apoptosis and autophagy through blocking the Akt/mTOR signaling pathway in various cancer cells (25–27). Thus, the Akt/mTOR pathway may be associated with both apoptosis and autophagy, and play a crucial role in SS-induced apoptosis and autophagy in gastric cancer cells. In our research, we observed that Akt/mTOR pathway was involved in autophagy and apoptosis triggered by SS, as evidenced by the downregulated expression of p-Akt and p-mTOR. Furthermore, inhibition of Akt/mTOR signaling pathway could directly activate autophagy and apoptosis (41, 42). In our study, inhibition of phosphorylated Akt or mTOR by specific inhibitor MK-2206 reinforced SS-induced apoptosis and autophagy in gastric cancer cells. These results indicated that SS induced apoptosis and autophagy in gastric cancer cells by blocking the Akt/mTOR signaling pathway. However, the in-depth mechanisms require to be explored in the future.

**Figure 7:**
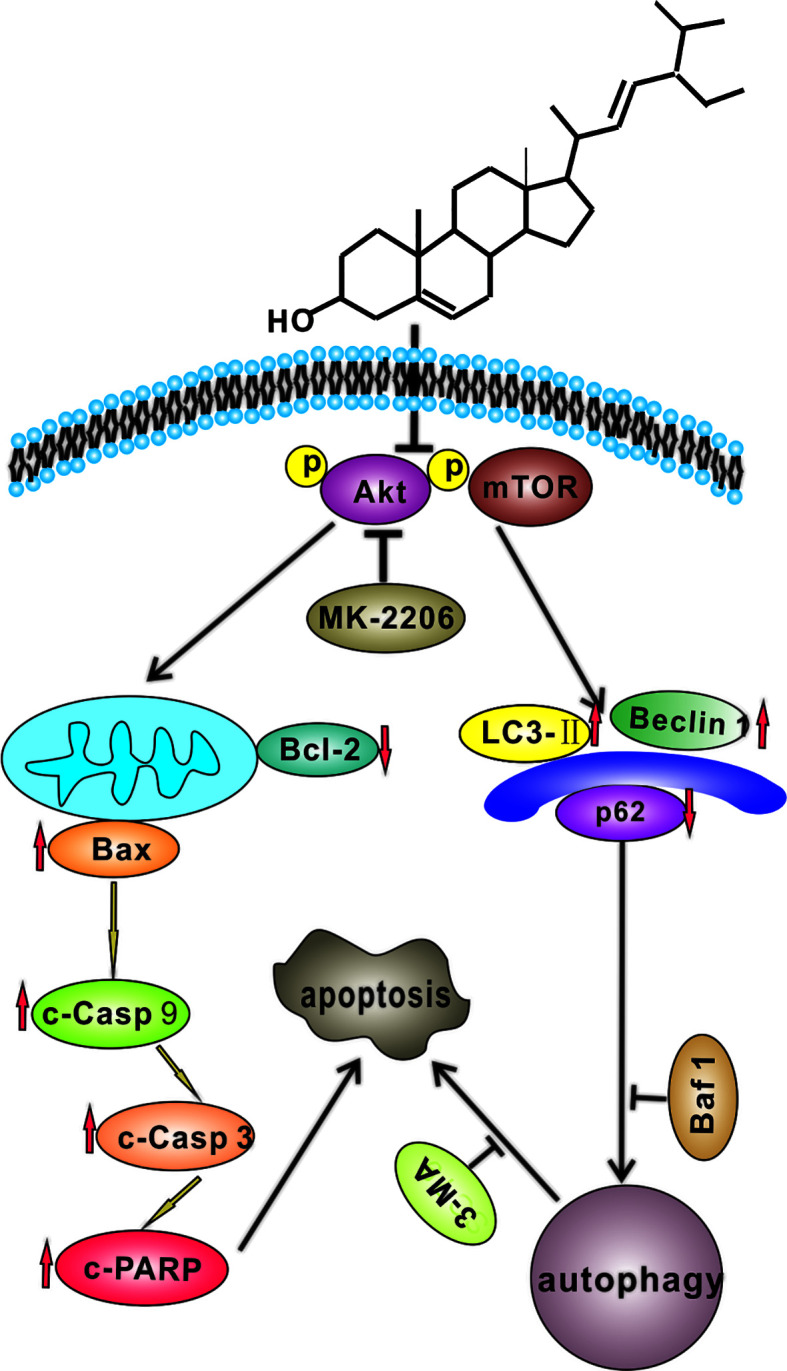
A hypothetical molecular mechanisms of stigmasterol (SS) induced anticancer properties in gastric cancer cells. SS simultaneously induces apoptosis and protective autophagy by inhibiting Akt/mTOR pathway in gastric cancer cells.

In conclusion, we demonstrated that SS could induce apoptosis in gastric cancer cells *in vitro* and inhibit cell growth *in vivo*. Furthermore, our study for the first time proved that SS could trigger apoptosis and protective autophagy through blocking Akt/mTOR pathway in gastric cancer cells. It would enrich our understanding of the antitumor properties of stigmasterol and provide evidence that stigmasterol may become a potential anticancer drug in treating gastric cancer in the future. We believe that our work provides new perspectives and holds potential for the development of stigmasterol. It will be of great interest, particularly to researchers working on oncology and nutrition. The underlying molecular mechanism of SS-induced gastric cancer cell death is exhibited in **Figure 7**. These results indicate that SS combined with autophagy inhibitor could be a promising therapy for gastric cancer.

## Data Availability Statement

The original contributions presented in the study are included in the article/supplementary material. Further inquiries can be directed to the corresponding author.

## Ethics Statement

The animal study was reviewed and approved by IRB of Hainan Medical University.

## Author Contributions

HZ: designed experiments. HZ and XZ carried out experiments. HZ and MW analyzed experimental results and interpretation. YL: data collection. SZ and HZ: data statistical analysis and writing. All authors contributed to the article and approved the submitted version.

## Funding

This work was supported by the Open Fund Project of Hainan Provincial Key Laboratory of Basic Medicine (grant JCKF2020009) and the Key Research and Development Project of Hainan Province (grant ZDYF2019177).

## Conflict of Interest

The authors declare that the research was conducted in the absence of any commercial or financial relationships that could be construed as a potential conflict of interest.
